# In Silico Study on Binding Specificity of Gonadotropins and Their Receptors: Design of a Novel and Selective Peptidomimetic for Human Follicle Stimulating Hormone Receptor

**DOI:** 10.1371/journal.pone.0064475

**Published:** 2013-05-20

**Authors:** Archana Sonawani, Sarfaraj Niazi, Susan Idicula-Thomas

**Affiliations:** Biomedical Informatics Center of Indian Council of Medical Research, National Institute for Research in Reproductive Health, Mumbai, India; University of Edinburgh, United Kingdom

## Abstract

Gonadotropins bind to specific receptors in spite of sharing a high level of sequence and structural similarity. This specific binding is crucial for maintaining the reproductive health of an organism. In this study, residues that dictate the receptor binding specificity of the gonadotropins (FSH and LH) have been identified using combination of in silico methods. Docking studies (ZDOCK), based on the systematic replacement of these residues, confirmed its importance in receptor binding. An interesting observation is that the relative positioning of the residues conferring binding specificity varied for the gonadotropin-receptor complexes. This spatial difference of the key residues could be exploited for design of specific modulators. Based on the identified residues, we have rationally designed a peptidomimetic (FSHP) that displays good binding affinity and specificity for hFSHR. FSHP was developed by screening 3.9 million compounds using pharmacophore-shape similarity followed by fragment-based approach. It was observed that FSHP and hFSHâ can share the same receptor binding site thereby mimicking the native hFSHR-FSH interactions. FSHP also displayed higher binding affinity to hFSHR as compared to two reported hFSHR antagonists. MD simulation studies on hFSHR-FSHP complex revealed that FSHP is conformationally rigid and the intermolecular interactions are maintained during the course of simulation.

## Introduction

G-protein coupled receptors (GPCRs) form the largest family of integral membrane proteins. These proteins are evolutionarily conserved and share a common seven α-helical transmembrane domain in their structures [1]–[3]. They are activated by a large number of diverse ligands such as small compounds, peptides or large proteins. The activated cell surface receptors transmit signals that induce a cellular response to the environment [4]. GPCRs are involved in many diseases and therefore are important drug targets [5]–[8].

The gonadotropin receptors, which include follicle-stimulating hormone receptor (FSHR) and lutropin/choriogonadotropin receptor (LH/CGR), belong to the class A group of GPCRs. The transmembrane domains (TMD) display high sequence identity (≈70%) whereas the ectodomains are less similar (≈40%). The gonadotropins viz., FSH, LH and CG are heterodimeric glycoprotein hormones. They have a common α-subunit and a hormone specific β-subunit [9], [10]. They are highly specific towards their cognate receptors, in spite of sharing high level of structural similarity. While FSH binds specifically to FSHR, the high sequence similarity (≈80%) in the β-subunits of LH and CG enables them to share a common receptor (LH/CGR). The binding of gonadotropins to their receptors initiates a signalling cascade which eventually brings about maturation of ovarian follicles in females and spermatogenesis in males [11]. These interactions are therefore crucial for regulating reproduction and gonadal development.

The extracellular domain (ECD) of the receptors and the β-subunit of the hormones has been experimentally shown to govern binding specificity [12], [13]. In the absence of the crystal structure of the complete hormone-receptor complex, several experimental studies using different approaches have been undertaken to delineate the residues important for binding specificity. In case of receptors, chimera studies have shown that β-strands 3 and 6 of human LHR (hLHR) are important for dictating the binding selectivity [14]. Alanine scanning and mutation studies for all the residues of these two β-strands revealed that residues 104N of β-strand 3 and 179G of β-strand 6 contribute to the selectivity of binding of hLHR to hLH/CG. Thus, introduction of residue 104N in hFSHR promotes hLH/CG binding to hFSHR whereas 179K of hFSHR prevents this binding [15]. Introduction of β-strand 1 of hFSHR into hLHR led to its binding to hFSH and introduction of a combination of β-strand 1 with few other β-strands of hFSHR into hLHR increased its binding affinity to hFSH. These observations suggested that residues belonging to multiple strands of hFSHR contribute towards binding selectivity [16].

Chimeric hCG/FSH β-subunits were constructed and analysed for their ability to interact with hLHR and hFSHR as well as hormone specific monoclonal antibodies. Substitution of 33–52 residues of hFSH with 39–58 residues of hCG showed no effect on receptor binding. However, substitution of 94–145 of hCGβ with 88–108 of hFSHβ resulted in a hormone analogue identical to hFSH in its ability to bind to hFSHR [17]. To further delineate the residues of this region that dictate binding specificity, 88DSDS91 and 95TVRGLG100 regions of hFSH were replaced by hLH residues 94RRST97 and 101GGPKDH106 respectively. The first substitution did not affect hFSHR binding but conferred hLHR binding to the chimera. The second substitution caused loss of binding to both hFSHR and hLHR. The study reveals that hFSH residues 95TVRGLG100 are crucial for FSH binding specificity whereas hLH residues 94RRST97 are involved in conferring hLHR binding specificity [18]. The results suggest that the regions/residues that contribute to binding specificity differ for the gonadotropins and several residues contribute to binding specificity. In such cases, where the binding surface is large, the knowledge of important residues involved in protein-protein interaction is exploited to design peptidomimetic modulators [19]–[21].

Various strategies have been used to design peptidomimetics, such as, insertion of unnatural amino acids, introduction of conformational constraints, isostearic replacement of peptide bonds [22], inversion of amino acid sequences and α-carbon chirality [23], screening and identification of suitable scaffolds based on shape and pharmacophore based similarity, followed by fragment-based approach [24]. Peptidomimetics have been successfully designed for various therapeutically important GPCR targets such as neuropeptide PLG analogs for modulating dopamine receptor [25], Pasireotide (SOM230) mimic somatostatin [26], Aba-Gly scaffold-based peptidomimetic forμ-opioid receptor [27], cyclic α-MSH analogs for melanocortin-4 receptor [28] and orally active GnRH antagonist AEZS-115 [29]. Efforts to design successful FSHR peptidomimetic modulators are still ongoing [30].

Since FSHR-FSH interaction is crucial for gonadotropin action, molecules that can block or mimic this interaction can serve as fertility regulating agents. This study aims to delineate the residues of gonadotropins and their receptors that influence binding specificity based on the available sequence and structural information and leverage this information for the rational design of FSH peptidomimetic. This is the first comprehensive structure-based study aimed at designing FSH peptidomimetics using the knowledge of hFSHR-FSHβ binding specific residues. The designed FSH peptidomimetic was found to have good binding affinity, stability and selectivity to hFSHR as assessed by docking followed by MD simulation studies. The information gained by this study could aid in the rational design of fertility regulating agents.

## Methods

### Dataset creation

Sequences of gonadotropins (FSH and LH) and their receptors (FSHR and LHR) found in various organisms were retrieved from Protein database of NCBI (Table 1). The sequence information was restricted to β-subunit of hormones and the ECD region of receptors.

**Table 1:** Sequence datasets of gonadotropins and their receptors.

Gonadotropins/Receptors	No. of sequences
FSH	21
LH	21
FSHR	16
LHR	9

### Structural analysis

The crystal structure of hFSHR-FSH was retrieved from Protein Data Bank (PDB ID: 1XWD), while the modeled, energy minimized structure of hLHR-LH was retrieved from Glycoprotein-hormone Receptors Information System database (GRIS) [31]. The modeled hLHR-LH complex structure was validated using Structural Analysis and Validation Server (SAVES, http://nihserver.mbi.ucla.edu/SAVES/). Recently, the entire ectodomain of hFSHR complexed with FSH has been elucidated (PDB ID: 4AY9). It is to be noted that the hormone-receptor interface of 4AY9 and 1XWD are identical. The β-subunit of hormones and ECD region of receptors were considered for analyses. The interactions that stabilize the hormone-receptor complex were identified using Protein Interactions Calculator (PIC) [32]. The hydrogen bonds and salt bridges were computed using HBPLUS [33] and Evaluating the Salt BRIdges in Proteins (ESBRI) [34] softwares respectively. The default parameters were used for identification of the interactions. The first and second shell residues of the interface region were considered as crucial for the hormone-receptor binding.

### Sequence analysis

The residues identified to be critical in stabilizing the hormone-receptor complex based on the structural analyses were evaluated for evolutionary conservation and uniqueness to the gonadotropin and the receptor family. Using in-house codes, the sequence information of gonadotropins and receptors was further simplified and reduced based on the physico-chemical properties of amino acids. The amino acids were grouped into neutral (N, Q, S, T), acidic (D, E), basic (K, R), aromatic (F, Y, W) and aliphatic (V, A, L, I, M) groups. C, P, H and G amino acids were considered independently.

BioEdit [35] version 5.0.9 was used to generate the pairwise and multiple sequence alignments (MSA). These alignments were validated by their corresponding structural alignments obtained using DSSP program [36].

### Generation of mutant structures

The in silico mutants were generated using Build Mutant protocol of Accelrys Discovery studio 2.0 (Acc. DS 2.0) and further energy minimized using 200 steps of steepest descent algorithm. The mutant hFSH (hFSHm) was generated by substituting all the residues of hFSH, identified to be important for hFSHR-FSH binding, with the corresponding residues of hLH. Mutant hFSHR (hFSHRm) was generated by substituting all the residues of hFSHR, identified to be important for hFSHR-FSH binding, with the corresponding residues of hLHR. Likewise, mutant hLH (hLHm) and mutant hLHR (hLHRm) were also generated (Table 2). The corresponding residue positions of the gonadotropins and their receptors were identified based on their sequence and structural alignments.

**Table 2:** Mutants created for gonadotropins and receptors.

hFSHRm	hLHRm	hFSHm	hLHm
E50R	A54V	P42V	R89S
R52S	Y55L	S89R	P97R
V54A	L101R	D90S	K98G
L55Y	Q103E	T95G	D105S
R101L	C128S	V96G	-
E103Q	Y178N	R97P	-
K104N	E199N	Y103T	-
K179G	-	-	-
V221K	-	-	-

### Molecular docking

#### Protein-protein docking

The structural coordinates of hFSHR-FSH and hLHR-LH were used for docking. The water molecules and hetero atoms were removed and the molecules were subjected to energy minimization using 200 steps of steepest descent algorithm. The energy minimised structures were docked using ZDOCK algorithm (Acc. DS 2.0).

For each of the docking simulations, 50 poses belonging to 10 clusters were generated. Clusters with maximum number of poses and/or whose cluster centre has Cα root mean square deviation (RMSD) within 10Å and RDOCK energy less than 10 Kcal/mol were shortlisted. The structural stability (based on the RDOCK energy) and presence of native-like interactions were used to identify the best pose from each of the shortlisted clusters, which were further used for structural analyses.

#### Peptidomimetic screening

GOLD [37], [38] v5.0 was used for initial screening of peptidomimetics retrieved from pepMMsMIMIC server [39]. The hFSHR (ECD) was prepared by removing hetero atoms and water molecules. Polar and non-polar hydrogen atoms, including those necessary to define the correct ionization and tautomeric states of residues were added. The binding site was defined by selecting Oε1 of 103E as the centroid atom and the binding site radius was set to 15 Å so as to encompass all the binding specific residues (BSRs). Default settings were used for docking the peptidomimetics. The top-ranked solutions obtained from each genetic algorithm run were then screened for identifying the peptidomimetic that shared maximum number of interactions with BSRs.

#### Protein peptidomimetic docking

Protein peptidomimetic docking simulations were performed using Glide Extra-Precision (XP) mode [40], [41]. The structures of the receptors and the peptidomimetics were prepared using Protein Preparation Wizard and LigPrep [42] v2.5 applications of Maestro [43] v9.2 respectively, using default settings. Receptor grids were generated for hFSHR using coordinates X = −1.970; Y = −23.420; Z = 30.930 and hLHR using coordinates X = −1.106; Y = −22.327; Z = 31.732. The structures were energy minimised using OPLS2005 forcefield. Poses were selected based on GlideScore, Model energy (Emodel) score and interactions with BSRs.

### Rational design of FSH Peptidomimetic

#### Pharmacophore-shape similarity based virtual screening of MMsINC® database

The 3D structure of FSHβ peptidic stretch 89S-103Y that includes the BSRs except 42P (as it is topologically distant) was used as the template for pepMMsMIMIC, a web-based peptidomimetic compound virtual screening tool. The pharmacophore model, generated based on the BSRs, was screened against a library of 17 million conformers obtained from 3.9 million commercially available chemical structures present in MMsINC® database [44]. The pepMMsMIMIC webserver employs five types of scoring methods to optimize the selection of the peptide mimetics, of which we have used viz., 1) fingerprint based filtering of shape similarity 2) based only on shape similarity and 3) based only on the pharmacophoric similarity. The 600 (top 200 per scoring method) peptidomimetics retrieved from pepMMsMIMIC were further screened by docking with hFSHR using GOLD. The best peptidomimetic identified by GOLD (MMs02514408) was further docked using Glide to assess the reproducibility of results.

#### Generation of novel peptidomimetics using fragment-based approach and optimization

The fragment-based approach for generation of novel peptidomimetics was carried out using the Fragmentor script, BREED and Glide tools available in Schrödinger.

MMs02514408 was fragmented using the Fragmentor script [45] and the fragments were docked with hFSHR using Glide. The BREED algorithm was used to hybridise the docked fragments and develop novel, chimeric molecules [46]. These chimeric molecules were then docked with hFSHR. The docked poses were analyzed with respect to their binding orientation, affinity and intermolecular interactions to identify the best peptidomimetic. The identified peptidomimetic was further modified by substituting solvent accessible groups by various functionalities to reduce the molecular weight and increase the binding affinity for hFSHR.

**Figure 1:**
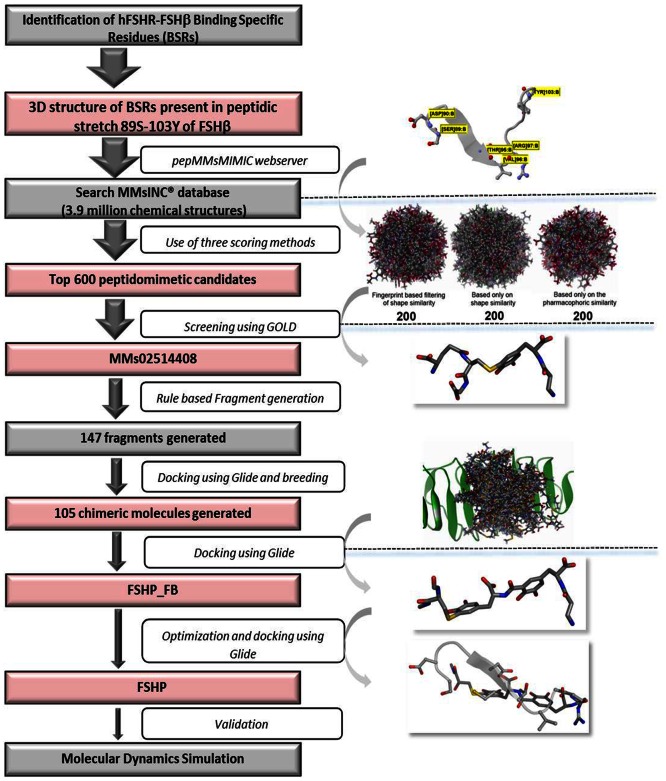
Flowchart describing the rational design of FSH peptidomimetic (FSHP). The structures represent information provided in pink boxes.

The twelve peptidomimetic derivatives thus generated, were then docked to hFSHR using Glide. The docked complexes were energy minimized using CHARMM forcefield with 200 cycles of conjugate gradient algorithm (DS 2.0). Generalized–Born was selected as an implicit solvent model while other parameters were kept default. The non-bonded interaction energies were specifically calculated between the BSRs and the docked peptidomimetics. Thus, the FSH peptidomimetic was designed and optimised using pepMMsMIMIC and fragment-based approach as shown in flowchart (Figure 1).

The structures were rendered using DS 2.0 and PyMol version 1.2 (http://www.pymol.org). Chemical structures were drawn using ChemDraw Ultra v8.0 [47].

### Molecular Dynamic Simulations

Molecular Dynamic (MD) simulations were performed for the docked complex of hFSHR-FSHP using GROMACS version 4.5.4 [48] with the implementation of the CHARMM forcefield [49]. The topology file for FSHP was generated by SwissParam using CHARMM all atoms forcefield [50]. The van der Waal interactions were calculated with a distance cut-off of 0.9 nm. Particle Mesh Ewald (PME) summation was applied for long range electrostatics with 1 nm cut-off for columbic interactions [51]. Counterions were added to neutralize the system. The system was solvated using SPC water model [52] and simulated in an octahedron box with periodic boundary conditions. The structures were first energy minimized using steepest descent algorithm with a tolerance of 1000 KJmol^−1^ nm^−1^. The system was equilibrated by applying position restraints on the complex and performing simulations using canonical NVT ensemble followed by NPT. Both the simulations were run for 100 ps each at a temperature of 300 K. Temperature coupling was performed using velocity rescaling [53] with a coupling constant of 0.1 ps and the initial velocities were generated according to Maxwell distribution. Temperature-pressure coupling was performed using extended-ensemble Parrinello-Rahman algorithm [54] with a coupling constant of 2 ps. The equilibrated system was then subjected to 5 ns of production run. A time step integration of 2 fs was used. The trajectories were saved every 500 steps and analysed using GROMACS analysis tools and XMGRACE-5.1.22 program (http://plasma-gate.weizmann.ac.il/Grace/).

## Results and Discussion

### Identification of residues important for binding specificity

The crystal structure of hFSHR-FSH complex is available in PDB (PDB ID: 1XWD, 4AY9) [55], [56] and the theoretical structure of hLHR-LH is available in the GRIS database. The quality of the hLHR-LH model was assessed using SAVES and it was found to be acceptable for further studies (See Table S1). The crystal structure of hFSHR (ECD)-FSHβ and the modeled structure of hLHR (ECD)-LHβ were analyzed to identify residues that contribute to the binding and stability of the hormone-receptor interactions. Since, the receptor residues that do not directly interact with the hormone i.e. the second shell residues could also influence hormone binding [57], the first and second shell residues in the hormone-receptor interface region were identified and the nature of their interactions was studied using PIC (Table 3).

**Table 3:** List of interactions that stabilize the gonadotropin-receptor complexes.

hFSHR Second* - First shell	Interaction	hFSHR First shell – hFSH^∞^	Interaction
*52R* - 50E	Electrostatic	50E - **97R**	Electrostatic
*54V* - 55L	Hydrophobic	55L - **103Y**	Hydrophobic
*103E* - 101R	Salt bridge	101R - **96V**	Hydrophobic
*103E* - 104K	Electrostatic	104K - **95T**	Hydrogen bond
-	-	179K - **89S**	Hydrogen bond
-	-	179K - **90D**	Salt bridge
*222I* - 221V	Hydrophobic	221V - **42P**	Hydrophobic
hLHR Second*- First shell	**Interaction**	**hLHR First shell – hLH^∞^**	**Interaction**
*54A* - 55L	Hydrophobic	55L - **105D**	Hydrogen bond
-	-	101L - **97P**	Hydrophobic
*128C* - 103Q	Hydrogen bond	103Q - **98K**	Hydrogen bond
-	-	178Y - **89R**	Cation-π
-	-	199E - **89R**	Electrostatic

* The second shell residues of the receptors are italicised. **^∞^**Interacting gonadotropin residues are represented in bold.

**Figure 2:**
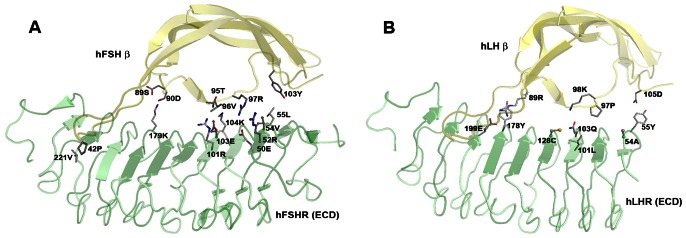
Binding specificity residues identified for (A) hFSHR-FSH complex and (B) hLHR-LH complex. The first shell residues are represented by sticks and the second shell residues are represented by ball and stick. The gonadotropins are colored yellow and the receptors are colored green.

The sequence information of gonadotropins and their receptors from various organisms were analysed to understand the degree of conservation for each of the residues identified from structural analysis. The corresponding residue positions of gonadotropins and their receptors were obtained from the sequence and structure-based alignments. The reduced alphabet representation of the sequences, based on the physicochemical properties of amino acids, helped to delineate residues that were strictly conserved and unique in the homologous sequences of a given family of gonadotropins and their receptors. For e.g. the basic residue (K/R) at position 97 is conserved in FSH while at the corresponding position, P is conserved in LH (Figure S1). As expected, the conservation of the physicochemical nature of residues of the gonadotropins is further reflected in the nature of the interacting residues of its cognate receptor. 97R of hFSHβ is involved in electrostatic interaction with 50E of hFSHR. 50E is stabilized by intra-molecular electrostatic interactions with 52R (second-shell residue) of hFSHR. Instead of an acidic residue (D/E) at position 50, which is conserved in FSHR, a basic residue (K/R) is conserved in LHR at the corresponding position. Similarly, other residues that are crucial for binding specificity were delineated based on structural analysis and sequence conservation (Figure 2 and Table 4).

**Table 4:** Binding specific residues identified for gonadotropin-receptor complexes.

hFSHR	hFSH	hLHR	hLH
50E	42P	55Y	89R
55L	89S	101L	97P
101R	90D	103Q	98K
104K	95T	178Y	105D
179K	96V	199E	-
221V	97R	*54A*	-
*52R*	103Y	*128C*	-
*54V*	-	-	-
*103E*	-	-	-

The second-shell residues are italicized.

### Validation of the BSRs by docking studies

Mutants for the gonadotropins and receptors were created based on the residues identified to be critical for binding specificity from sequence and structural analysis. The mutant and wild-type structures of the gonadotropins and their receptors were docked and the energies of the complexes compared to understand the contribution of the identified residues to binding stability. The docking algorithm was initially validated by re-docking the wild-type structures, which correctly reproduced the X-ray crystal structure of hFSHR-FSH (1XWD) and the modeled structure of hLHR-LH with RMSD of 0.98Å and 1.3Å respectively (Figure S2).

ZDOCK generated eight to ten clusters containing several docked poses for each of the 6 complexes and ranked them according to their ZDock score. Subsequent to RDOCK refinement, clusters were short-listed for each of the docked complexes based on the selection/filtering criteria (see methods). The best pose within each of the selected cluster centers were then identified based on RDOCK energy and presence of native-like interactions. The docking studies revealed that the stability of the wild type complexes is remarkably higher than the mutants (Table 5). This observation validates the role of the identified residues in stabilizing the gonadotropin-receptor interactions.

**Table 5:** RDOCK energy of the docked complexes.

Docked complex	Energy (Kcal/mol)		Docked complex		Energy (Kcal/mol)		
hFSHR-hFSH		−34.16		hLHR-hLH		−30.47	
hFSHR-hFSHm		14.3		hLHR-hLHm		−12.0	
hFSHRm-hFSH		23.4		hLHRm-hLH		10.3	

### Observed cross-reactivity of gonadotropins could be explained by BSRs

In spite of the interactions between gonadotropins and their receptors being highly selective within species, there have been reports of gonadotropin-receptor cross-reactivity observed amongst different species. For e.g. horse LH can bind to rat and all mammalian FSHRs [58], [59], chicken LH binds with higher affinity to rat FSHR as compared to rat LHR [60] and hFSHR can be activated by porcine and bovine FSH at higher concentrations [61]. We sought an explanation to the first two cases of reported cross-reactivity of gonadotropins and receptors based on the residues that have been identified as important for binding specificity by our study.

We have identified that the presence of an aliphatic residue at 96 position of FSH is crucial for FSHR interaction (Figure 2). It is noteworthy that horse LH has 96V (instead of 96G that is conserved amongst LH) and probably for this reason, horse LH can bind to FSHR of other mammalian species (Figure S1). The observation that chicken LH binds with higher affinity to rat FSHR than rat LHR could be explained based on the following: a) the presence of a basic residue at 179 position of FSHR is important for FSH binding (Figure 2). Interestingly, chicken LHR also has a basic residue, lysine at position 179 instead of the conserved glycine seen at 179 position of LHR; b) Proline at 42 position of FSH is crucial for FSHR binding (Figure 2). Chicken LH has 42P instead of leucine which is conserved in LH.

Although these observations might give a plausible explanation to the observed cross-reactivity, further studies are to be carried out to understand whether single residue or synergistic co-operativity of multiple residues are necessary to generate the binding specificity that operates in gonadotropins and their receptors. It is highly likely that the crucial function of binding specificity of gonadotropins has been entrusted to multiple residues.

### Peptidomimetics designed based on BSRs

#### Pharmacophore-shape similarity based approach for identification of suitable FSH peptidomimetics

**Figure 3:**
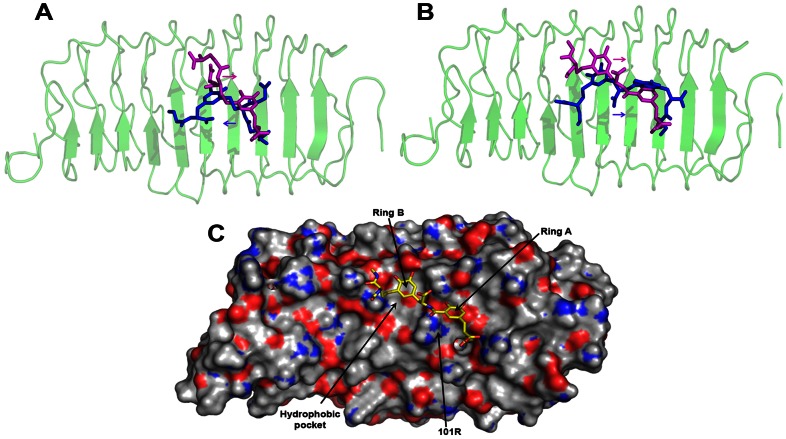
Binding modes of MMs02514408 and FSHP_FB predicted by GOLD and Glide. (A) Opposite binding modes predicted for MMs02514408 with hFSHR (B) Similar binding modes predicted for FSHP_FB with hFSHR. The GOLD and Glide docked poses are shown as blue and pink sticks respectively. hFSHR is depicted in green cartoon representation. (C) Figure illustrating the importance of two planar hydrophobic groups (phenyl rings A and B) present in FSHP_FB (yellow sticks) for hFSHR binding (Molecular surface representation). Ring A forms cation-π interaction with 101R while ring B is embedded in the hydrophobic pocket of the binding site.

A pharmacophore model was generated based on the identified BSRs of hFSHβ using pepMMsMIMIC web server. This model was used to retrieve peptidomimetic candidates from 3.9 million commercially available chemical structures present in MMsINC® database (see Methods). From the retrieved 600 molecules, the peptidomimetic, MMs02514408 was selected since it displayed a) maximum pharmacophoric similarity to the BSRs; b) best docked solution with hFSHR and c) maximum interactions with BSRs of hFSHR (Figure S3). Comparison of the best docked solutions obtained using GOLD and Glide indicated opposite binding modes of MMs02514408 with hFSHR (Figure 3A). Examination of the overlaid docked poses revealed that all the BSRs (except 179K) interacted with MMs02514408 (See Table S2).

#### Generating novel molecules using fragment-based approach

Fragment-based approach was adopted to generate novel molecules with higher binding affinity to hFSHR. MMs02514408 was fragmented and docked to hFSHR using Fragmentor script and Glide of Schrödinger. The docked fragments were subjected to the popular BREED algorithm to generate 105 novel molecules (see Methods). The best peptidomimetic (FSHP_FB) was selected based on the analysis of the docked poses of these molecules with hFSHR. FSHP_FB was selected since it displayed high binding affinity for hFSHR and interacted with all BSRs including 179K (See Table S3).

Interestingly, unlike MMs02514408, both GOLD and Glide docking algorithms predicted a similar binding orientation of FSHP_FB with hFSHR (Figure 3B). The differential positioning of the aromatic ring of MMs02514408, as observed in the superpositioned GOLD and Glide docked poses, reveal the presence of two hydrophobic ring binding centres on hFSHR. Incidentally, FSHP_FB has two aromatic rings separated by conformationally constrained spacer (discussed later). Ring A of FSHP_FB forms cation-π interaction with 101R of hFSHR while ring B is embedded in the hydrophobic pocket of the binding site (Figure 3C). The presence of two phenyl ring binding centres on hFSHR and the increased binding affinity displayed by FSHP_FB as compared to MMs02514408 pinpoints the positive contribution of the two phenyl rings of the ligand for enhanced binding affinity to hFSHR.

#### Optimisation and development of FSH peptidomimetic (FSHP)

**Figure 4:**
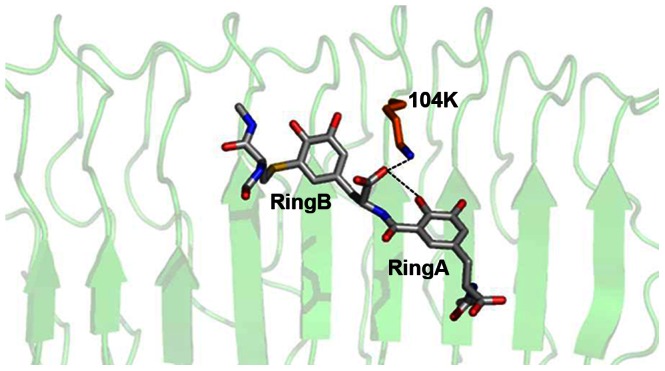
Inter and intra molecular interactions provide rigidity to the spacer connecting rings A and B. Anionic carboxyl oxygen atom of FSHP_FB (gray sticks) is involved in intramolecular hydrogen bond formation with hydroxyl group present at the *ortho-*position of Ring A and also forms salt bridge with 104K (orange stick) of hFSHR (green cartoon). The interactions are shown in black dotted lines.

FSHP_FB has three stereocentres with R-configuration viz., 11*Cα_1_*-1, 21*Cα_2_*-2 and 53*Cα_3_*-3. The configuration of the first stereo centre is similar to L-cysteine while the latter two are similar to D-phenylalanine. The solvent accessible formamide (at 11*Cα_1_*) and carboxyl (at 53*Cα_3_*) groups were modified to reduce the conformational flexibility and molecular weight. The carboxyl group at 21*Cα_2_* position was not modified since its anionic oxygen is involved in salt bridge formation with cationic nitrogen of 104K of hFSHR. Additionally, the oxygen is also involved in intra-molecular H-bonding with hydroxyl group at the *ortho*-position of phenyl ring A in FSHP_FB. These interactions assist in reducing the conformational flexibility of the phenyl rings of FSHP_FB (Figure 4).

**Figure 5:**
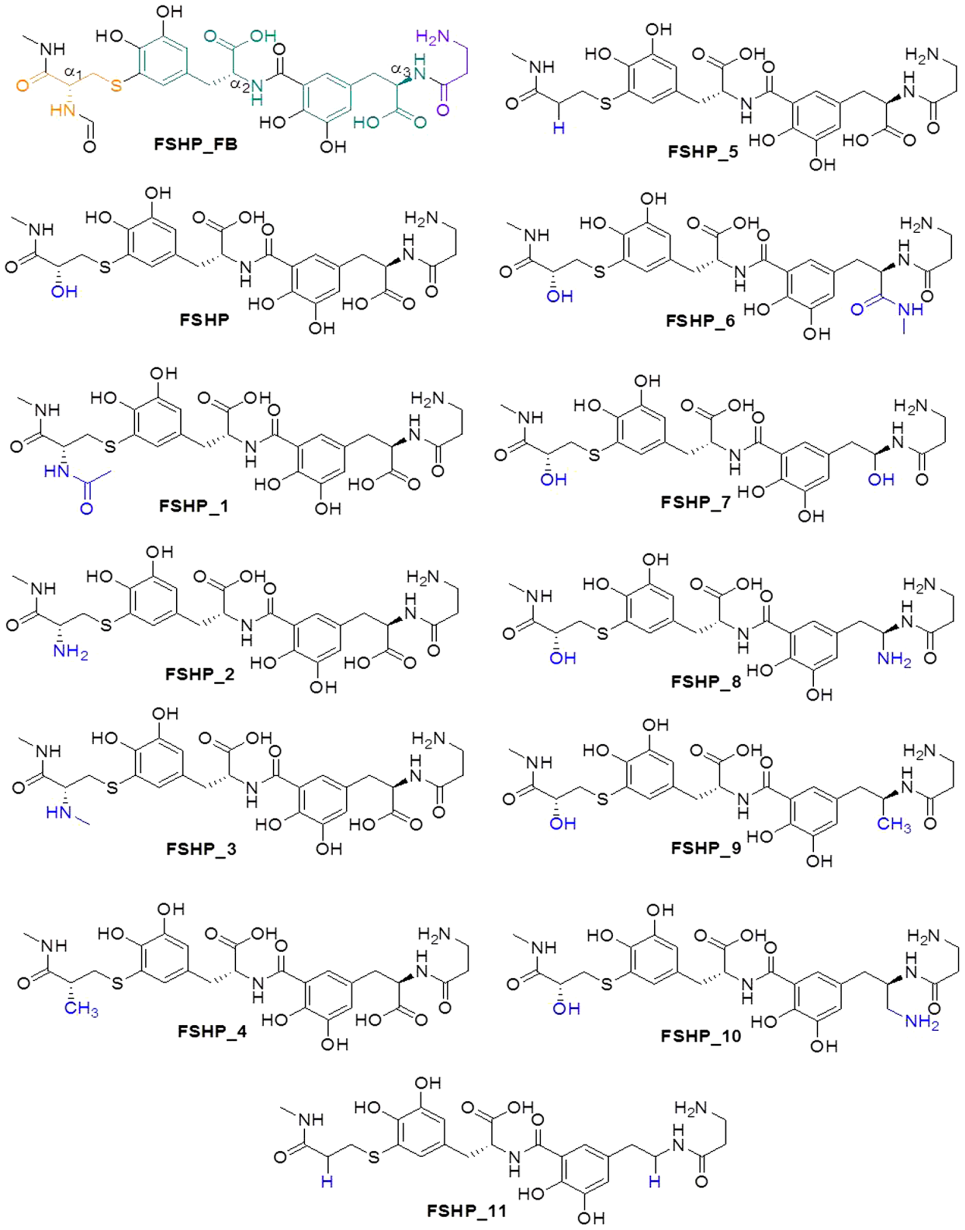
Chemical structures of the peptidomimetics obtained by in silico structural modifications made at two stereo centers of FSHP_FB viz. , 11*Cα_1_* and 53*Cα_3_*. Substituent groups/atoms are highlighted in blue. FSHP_FB has four structural units viz., L-cysteine (orange), D-phenylalanine (green) and β-alanine (purple).

Twelve peptidomimetics were generated by substituting the solvent accessible formamide (at 11*Cα_1_*) and carboxyl (at 53*Cα_3_*) groups with different functionalities (Figure 5). These peptidomimetics were docked with hFSHR. The peptidomimetic, FSHP displayed maximum number of interactions with BSRs and exhibited favourable GlideScore, Emodel and non-bonded interaction energies, which were specifically calculated between the BSRs and the docked peptidomimetics (Table 6). The hydroxyl group at 11*Cα_1_* position of FSHP mimics 89S of hFSHβ and engages in native-like interaction with 179K of hFSHR as observed in the docked complex.

**Table 6:** Docking of peptidomimetics generated during optimisation of FSHP_FB.

Peptidomimetic§	Interacting BSRs of hFSHR	GlideScore (Kcal/mol)	EModel	Interaction Energy (Kcal/mol)
FSHP_FB	50E, 101R,103E 104K,179K	−7.83	−67.44	−180.86
FSHP	52R, 101R, 103E, 104K, 179K	−7.98	−75.40	−180.90
FSHP_1	50E, 101R,103E, 104K	−7.83	−67.44	−184.06
FSHP_2	50E, 103E, 104K	−8.62	−82.54	−110.81
FSHP_3	50E, 101R, 103E	−6.83	−73.42	−108.22
FSHP_4	50E, 103E, 104K	−8.88	−77.22	−163.14
FSHP_5	50E, 101R	−7.73	−63.96	−118.31
FSHP_6	50E, 103E,104K, 179K	−6.82	−79.06	−145.94
FSHP_7	50E, 52R	−6.35	−69.42	−49.12
FSHP_8	50E, 104K	−7.96	−86.92	−85.04
FSHP_9	50E, 103E	−5.88	−67.53	−104.00
FSHP_10	103E, 104K	−7.71	−75.32	−81.62
FSHP_11	50E, 101R,103E	−6.70	−76.83	−52.16

§ Chemical structures of the peptidomimetics have been shown in Figure 5.

The presence of carboxyl (at 53*Cα_3_*) group in FSHP constrains the 57*N*-53*Cα*-52*Cβ*-45*Cγ* dihedral angle to energetically favourable gauche-(−), 72.5° conformation (in case of FSHP_FB, the dihedral angle was gauche-(+), 60°). This dihedral angle is critical for placement of the terminal cationic nitrogen (N70) of FSHP in a manner that can mimic the 97R BSR of hFSHβ. This conformation also facilitates cation-π interaction between 101R of hFSHR and phenyl ring A of FSHP (Table 7, Figure S4).

**Table 7:** Interactions between FSHP and hFSHR in the docked complex.

hFSHR∞	FSHP^ţ^	Interaction
*104K(Nζ)*	O51	H bond
*104K(Nζ)*	O41	Electrostatic
*101R(Nη_2_)*	O28	H bond
*101R(Nη_2_)*	Phenyl ring-A	Cation-π
*179K(Nζ)*	O17;O14	H bond
*103E(Oε_1_)*	O51	H bond
*52R (Cδ)*	C61	Hydrophobic
*52R (Cδ)*	C62	Hydrophobic
76E(Oε_1_)	N74	Electrostatic
76E(Oε_2_)	O50	H bond
78S(Oγ)	O50	H bond
128S(Oγ)	O7	H bond
152Q(NεH_1_)	S9	H bond
153D(Oδ_2_)	O8	H bond
153D(Oδ_2_)	O17	H bond
153D(Oδ_1_)	N15	H bond

∞ BSRs are italicised. ^ţ^ Refer to Figure S4B for structural details.

### MD simulation studies confirms stability of the hFSHR-FSHP complex

**Figure 6:**
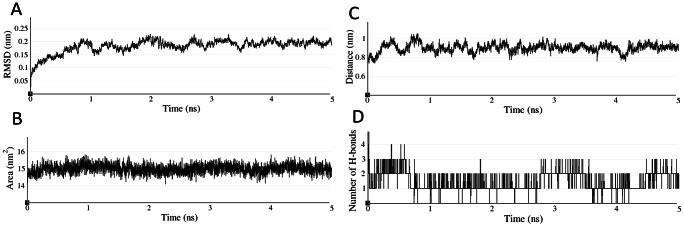
MD trajectory analysis of hFSHR-FSHP complex. (A) Backbone RMSD (B) Total SASA^ξ^ (C) Distance^ξ^ and (D) Number of H-bonds^ξ^. ^ξ^Calculated between the BSRs of FSHR and FSHP.

The stability of the hFSHR-FSHP docked complex was assessed by performing MD simulations for 5 ns. The RMSD of the backbone atoms was calculated with respect to the starting structure as a function of time. The complex achieved stability with an RMSD value around 0.17 nm within 1 ns (Figure 6A). The radius of gyration indicated that the structures persisted in the bound state and fluctuated marginally by 0.3Å relative to the initial structure (Figure S5c). The potential energy plotted as a function of time was found to be stable beyond 0.3 ns (Figure S5d).

We analysed the trajectory for total solvent accessible surface area (SASA), distance and number of hydrogen bonds between BSRs of hFSHR and FSHP (Figures 6B-D). SASA and the distance between FSHP and the BSRs (∼ 0.9 nm) did not fluctuate significantly during the course of simulation. We also noticed that FSHP primarily formed 2 hydrogen bonds throughout the simulation with maximum of 4 hydrogen bonds as observed in the docked pose. The above analysis confirms that FSHP stably occupies the binding site of hFSHR throughout the course of simulation.

**Figure 7:**
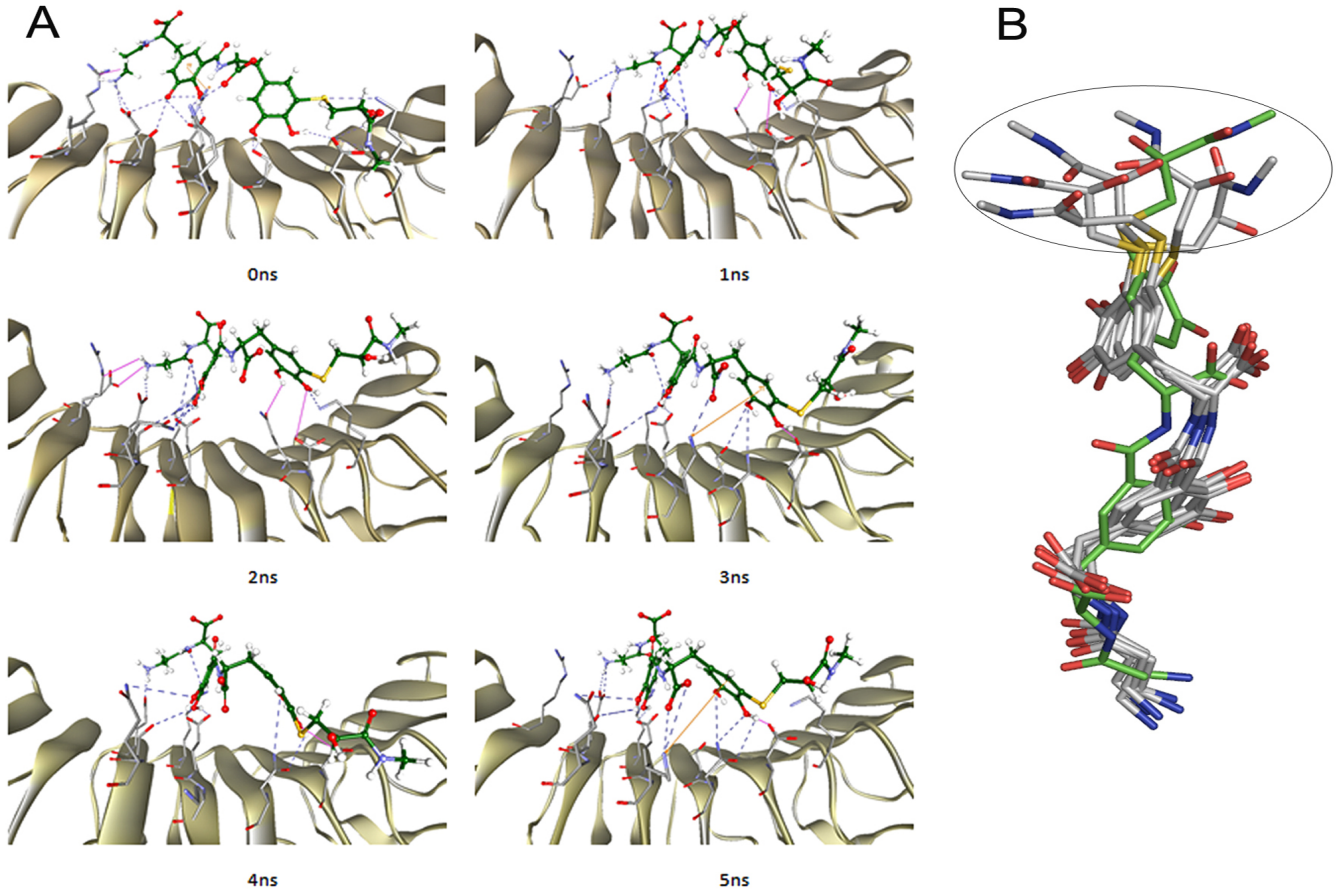
Snapshots of hFSHR-FSHP complex during the course of MD simulation. (A) The binding mode and interactions of FSHP (green ball and sticks) with hFSHR (cartoon) shown at different time intervals. The hydrogen bonds, electrostatic and cation-π interactions are shown in blue, pink and orange lines respectively. (B) Molecular overlay of FSHP conformations taken at intervals of 1 ns. The conformation of FSHP taken at 0 ns is shown in green sticks. The region of FSHP that displays flexibility during the simulation is circled.

The average RMS fluctuation was plotted to estimate the extent of residue-wise fluctuations in the hFSHR interface region when bound to FSHP (Figure S5b). During the entire course of simulation, all the BSRs showed minimal fluctuations within the range of 0.05–0.1 nm (Figure S5a). The low RMS fluctuations indicate that the binding region is rigid and the complex is tightly bound. Snapshots of the intermolecular interactions of hFSHR-FSHP taken at 1 ns intervals of MD simulation are shown in Figure 7A. The overlay of the six representative FSHP conformations reveals that the structure is conformationally rigid except at the 2-hydroxy-N-methyl-propanamido termini (Figure 7B).

**Figure 8:**
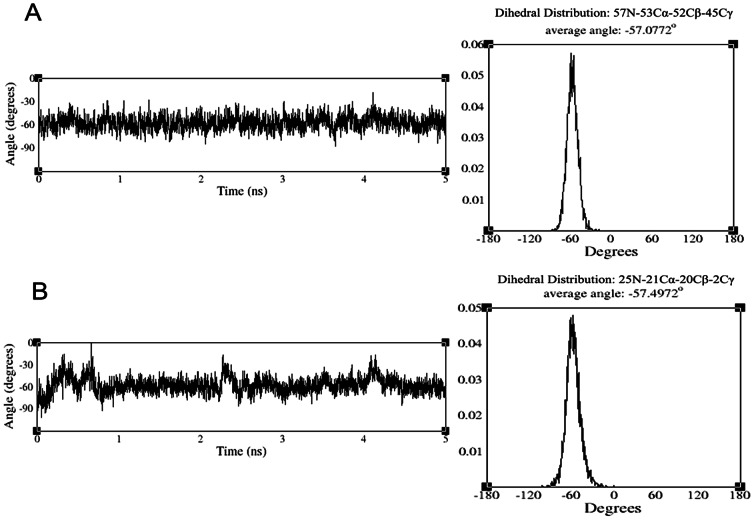
Analysis of two dihedral angles of FSHP during MD simulation. The average dihedral angles and probability distributions plotted for (A) 57N-53Cα-52Cβ-45Cγ (B) 25N-21Cα-20Cβ-2Cγ.

To quantify the conformational flexibility of FSHP when bound to hFSHR, we evaluated the distribution of two dihedral angles viz., 57*N*-53*Cα*-52*Cβ*-45*Cγ* and 25*N*-21*Cα*-20*Cβ*-2*Cγ* which form an important part of the backbone scaffold of FSHP during the course of MD simulation. Dihedral transitions were absent throughout the simulation and the average dihedral angles for 57*N*-53*Cα*-52*Cβ*-45*Cγ* and 25*N*-21*Cα*-20*Cβ*-2*Cγ* were found to be gauche-(−), 57.07° and 57.49° respectively (Figures 8A and B). This confirms that FSHP is conformationally rigid and the carboxyl groups at 21*Cα_2_* and 53*Cα_3_* positions help in constraining the angles in an energetically favourable conformation.

### FSHP has distinct pharmacophoric similarity to hFSHβ

**Figure 9:**
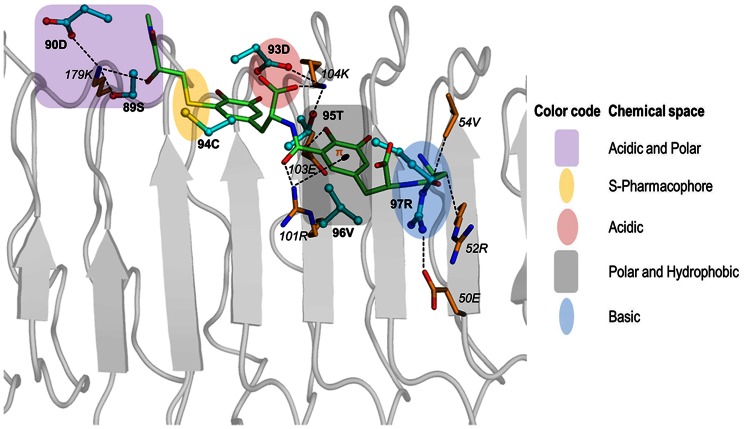
Pharmacophoric similarity of FSHP and hFSHβ. The chemical space shared by hFSHβ (cyan sticks) and FSHP (green sticks) is color-coded as explained in the side panel. The interactions with the BSRs (orange sticks) of hFSHR (grey cartoon) are shown in black dotted lines. FSHP shares similar pharmacophoric features and molecular interactions with BSRs of hFSHR as hFSHβ.

Examination and comparison of the bound structures of hFSHR-FSH and hFSHR-FSHP indicate that FSHP has distinct pharmacophoric similarity to the binding site of hFSHβ. The docked structures reveal that all the pharmacophoric functionalities of FSHP mimic the physicochemical properties of the BSRs of hFSHβ; such as, i) hydroxyl group at 11*Cα_1_* position mimics 89S; ii) pyrocatechol group (ring A) mimics 95T and 96V and iii) terminal alkyl amino group mimics 97R. FSHP has pharmacophoric groups that also mimic the neighbouring residues of BSRs of hFSHβ, such as carboxyl group at 21*Cα_2_* position and sulfide group of FSHP mimics 93D and 94C of hFSHβ respectively (Figure 9).

This pharmacophoric similarity explains the high binding affinity of FSHP to hFSHR, as observed from docking and MD studies and also validates its proposed FSH peptidomimetic function.

### FSHP specifically mimics the binding mode of hFSHβ and not hLHβ

**Figure 10:**
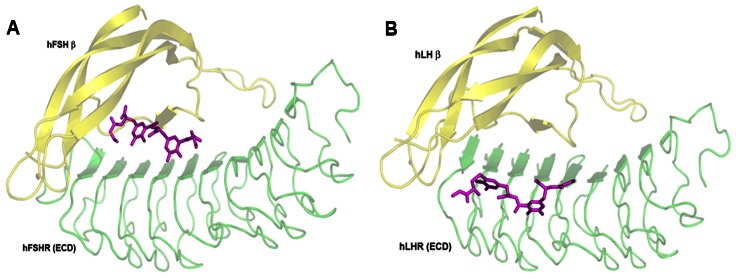
Differential binding modes of FSHP to hFSHR and hLHR. Structures of gonadotropins (yellow cartoon) complexed with their cognate receptors (green cartoon) are super positioned with docked FSHP (pink sticks)-receptor complexes. (A) FSHP binds to hFSHR in a similar mode as that of hFSHβ (B) FSHP binds to hLHR at a site distant from the hLHβ binding site.

Comparison of the results obtained from docking FSHP to hFSHR and hLHR revealed that while FSHP mimics the binding mode of hFSHβ, it distinctly differs with respect to the binding mode adopted by hLHβ (Figure 10). FSHP interacts with only one BSR of hLHR (178Y) as compared to five BSRs of hFSHR. This is not surprising as FSHP was designed based on the BSRs of hFSH. These results indicate that FSHP specifically mimics hFSH and not hLH.

### FSHP has better binding affinity to hFSHR as compared to two known hFSH antagonists

**Figure 11:**
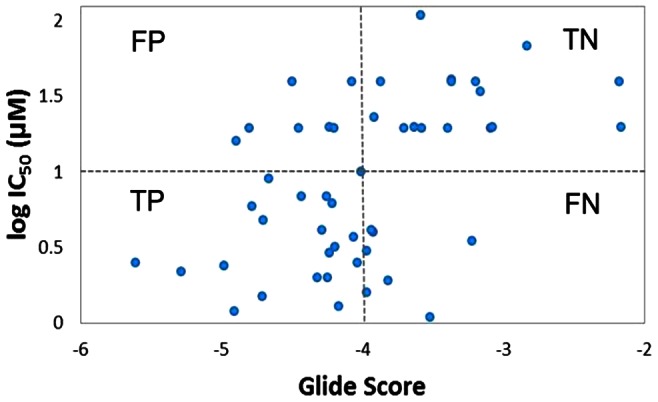
Plot of experimentally determined log IC50 values of 50 compounds versus their Glide scores.

**Figure 12:**
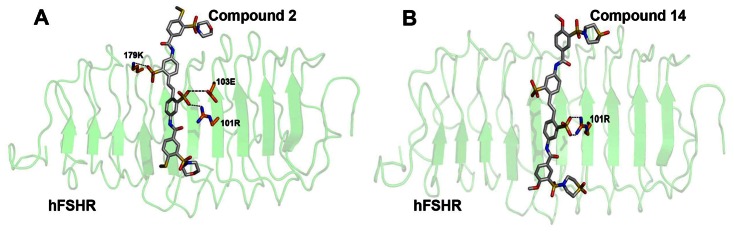
Docked complexes of hFSHR with (A) Compound 2 (B) Compound 14.

The docking protocol was validated using compounds that were experimentally tested for their potential to bind to the ECD region of hFSHR [62]. 50 such compounds with experimentally determined IC50 values were docked with the ECD region of hFSHR using Glide. Of the 50, 27 compounds had an IC50 value of <10 µM and were considered as actives (True positives) and the remaining 23 compounds were considered as inactives (True negatives). 20 of the 27 actives and 16 of the 23 inactives were predicted correctly as True positives and True negatives respectively. 7 of the actives and 7 of the inactives were wrongly predicted as False negatives and False positives respectively (Figure 11, see Table S4). The sensitivity and specificity of the docking protocol was found to be ∼0.7. Of the 27 actives, three FSH antagonists viz. Compounds 2, 14 and 50 were reported to bind to the ECD region of hFSHR with high affinity and selectivity of which Compounds 2 and 14 exhibited least IC50. Compounds 2 and 14 docked, as expected, to the binding site and exhibited GlideScores of −4.90 Kcal/mol (experimental IC50 1.2 µM) and −4.32 Kcal/mol (experimental IC50 2.0 µM), respectively as compared to a more favourable GlideScore of -7.98 Kcal/mol for FSHP. The docked poses of both compounds also showed lower number of interactions with BSRs as compared to FSHP (Figure 12).

## Conclusions

The residues crucial for influencing the binding specificity of gonadotropin-receptor complexes have been delineated by sequence and structural analysis. The importance of the identified residues for binding specificity was validated by protein-protein docking with systematic replacement of identified residues and rationalising few examples of cross-reactivity reported in literature for these gonadotropins. The 3D structural information of BSRs of hFSHβ was used to design a novel and selective FSH peptidomimetic (FSHP, Table 8) using pharmacophore-shape similarity and fragment-based approach.

**Table 8:** Chemical names of the designed peptidomimetics.

Peptidomimetic	Chemical Name
MMs02514408	(S)-2-amino-5-((R)-3-(5-((R)-2-(3-aminopropanamido)-2-carboxyethyl)-2,3-dihydroxyphenylthio)-1-(carboxymethylamino)-1-oxopropan-2-ylamino)-5-oxopentanoic acid
FSHP_FB	(R)-2″-(5′-((R)-2″-(3-aminopropanamido)-2″-carboxy-ethyl)-3″-(2′,3′-dihydroxybenzamido)-3-(3,4-dihydroxy-5-((R)-2-formamido-N-methyl-3-thio-propanamido)-phenyl)-propanoic acid
FSHP	(R)-2″-(5′-((R)-2″-(3-aminopropanamido)-2″-carboxy-ethyl)-3″-(2′,3′-dihydroxybenzamido)-3-(3,4-dihydroxy-5-((R)-2-hydroxy-N-methyl-3-thio-propanamido)-phenyl)-propanoic acid

While docking results reveal that FSHP can effectively mimic the chemical space of hFSHβ; MD simulations of hFSHR-FSHP complex have shown that FSHP stably interacts with BSRs of hFSHR. Molecular overlay of FSHP conformations, taken at 1 ns intervals, and the conformational analysis of two important dihedrals of FSHP indicate that FSHP is conformationally rigid except at the 2-hydroxy-N-methyl-propanamido termini connected to phenyl sulfanyl group (Figure 7B). Docking results confirm that FSHP specifically mimics hFSHβ and not hLHβ. It also has better binding affinity to hFSHR as compared to two known hFSH antagonists. Experimental studies are warranted to confirm the usefulness of FSHP, which is hypothesised to be a good lead candidate for development of fertility regulating agents.

## Supporting Information

Figure S1 **Conservation of physicochemical properties of residues involved in binding specificity.** 97R of hFSHβ (yellow) is involved in intermolecular interactions with 50E of hFSHR (green). 50E is stabilized by intra-molecular interaction with second shell residue 52R of hFSHR. (A) MSA for FSHβ representing the conserved basic residue (K, R) at position 97. (B) MSA for LHβ representing the conservation of P corresponding to 97^th^ residue of FSHβ.
(TIF)

Figure S2 **Validation of ZDOCK algorithm by redocking wild type structures of gonadotropin-receptor complexes.** (A) Superposition of X-ray crystal structure of hFSHR (green)-FSH (yellow) with the best pose obtained after re-docking (orange) (B) Superposition of modeled structure of hLHR (green)-LH (yellow) with the best pose obtained after re-docking (orange).
(TIF)

Figure S3 (A) Schematic and (B) Chemical structure of MMs02514408, chemical name; (S)-2-amino-5-((R)-3-(5-((R)-2-(3-aminopropanamido)-2-carboxyethyl)-2,3-dihydroxyphenylthio)-1-(carboxymethylamino)-1-oxopropan-2-ylamino)-5-oxopentanoic acid.
(TIF)

Figure S4 Schematic representations of (A) FSHP_FB and (B) FSHP.
(TIF)

Figure S5 **Analysis of MD trajectory.** (a) Histogram showing the RMSF values for BSRs. (b) RMSF plot showing residue-wise fluctuations of hFSHR (c) Radius of gyration (d) Potential energy.
(TIF)

Table S1 SAVES results for structural validation of hLHR-LH model.
(DOC)

Table S2 Interactions between MMs02514408 and hFSHR in the docked complexes.
(DOC)

Table S3 Interactions between FSHP_FB and FSHR in the docked complexes.
(DOC)

Table S4 Compounds with their experimental IC50 values and Glide Scores on binding with hFSHR.
(DOC)
