# Correction: In Silico Study on Binding Specificity of Gonadotropins and Their Receptors: Design of a Novel and Selective Peptidomimetic for Human Follicle Stimulating Hormone Receptor

**DOI:** 10.1371/annotation/bc97a85c-1ecd-4cd8-ab61-0aef01f949a1

**Published:** 2013-11-08

**Authors:** Archana Sonawani, Sarfaraj Niazi, Susan Idicula-Thomas

Several errors were introduced in the preparation of this article for publication.

There is an error in the eighth sentence of the Abstract. hFSHâ should be replaced by hFSHβ. The correct sentence reads: It was observed that FSHP and hFSHβ can share the same receptor binding site thereby mimicking the native hFSHR-FSH interactions.

There is an error in the footnotes of Table S3. The Interaction energy is incorrectly listed as 180.86 Kcal/mol. The correct Interaction energy is -180.86 Kcal/mol. 

