# Supplementary figures and images for: In Silico Study on Binding Specificity of Gonadotropins and Their Receptors: Design of a Novel and Selective Peptidomimetic for Human Follicle Stimulating Hormone Receptor
Source: PLoS One. 2013 May 20;8(5):e64475. doi: 10.1371/journal.pone.0064475 (PMC3659097; doi:10.1371/journal.pone.0064475)

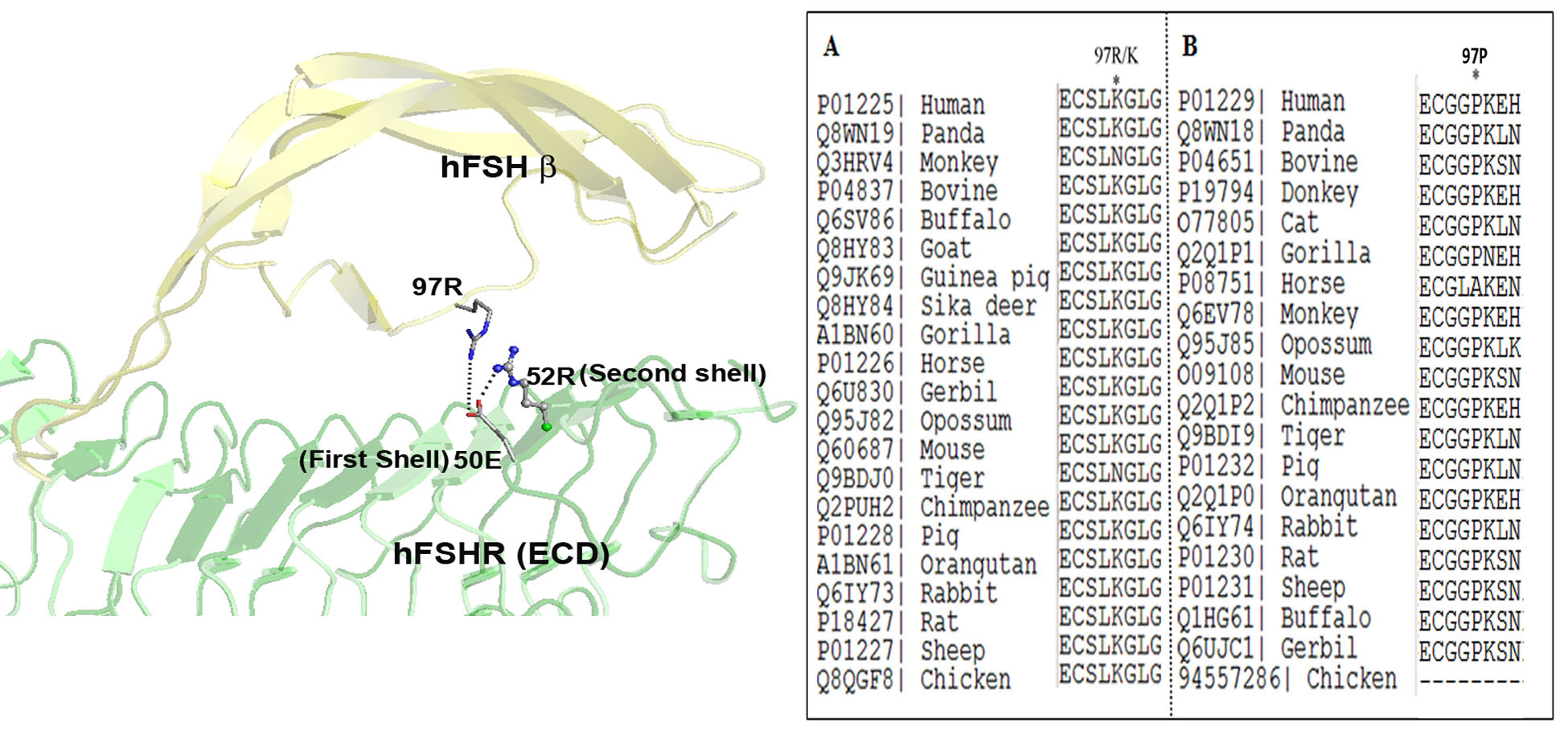

Supplement: Figure S1 — Conservation of physicochemical properties of residues involved in binding specificity. 97R of hFSHβ (yellow) is involved in intermolecular interactions with 50E of hFSHR (green). 50E is stabilized by intra-molecular interaction with second shell residue 52R of hFSHR. (A) MSA for FSHβ representing the conserved basic residue (K, R) at position 97. (B) MSA for LHβ representing the conservation of P corresponding to 97th residue of FSHβ. (TIF) [file pone.0064475.s001.tif]

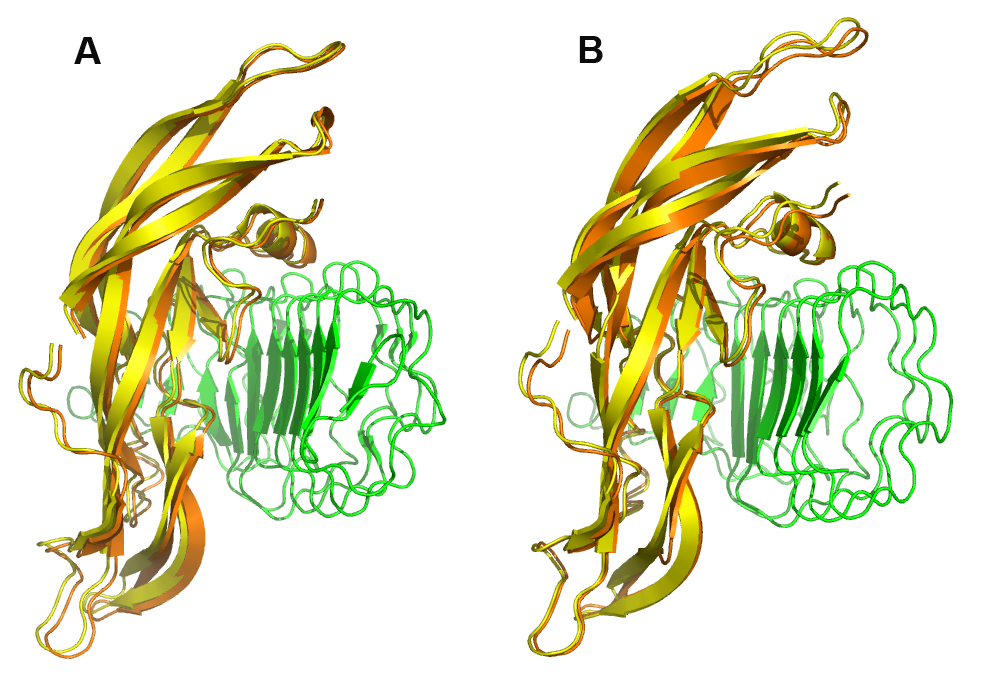

Supplement: Figure S2 — Validation of ZDOCK algorithm by redocking wild type structures of gonadotropin-receptor complexes. (A) Superposition of X-ray crystal structure of hFSHR (green)-FSH (yellow) with the best pose obtained after re-docking (orange) (B) Superposition of modeled structure of hLHR (green)-LH (yellow) with the best pose obtained after re-docking (orange). (TIF) [file pone.0064475.s002.tif]

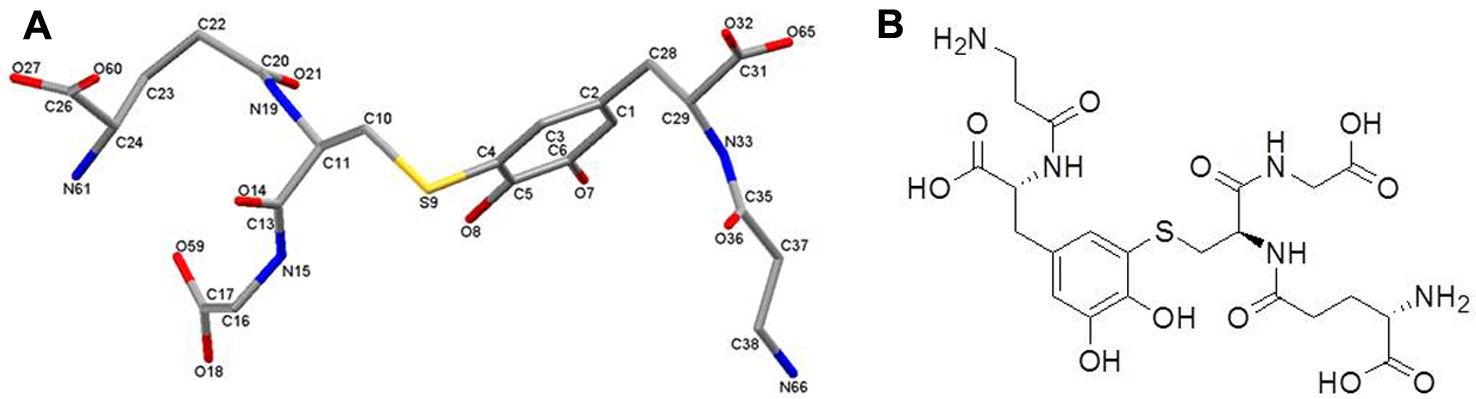

Supplement: Figure S3 — (A) Schematic and (B) Chemical structure of MMs02514408, chemical name; (S)-2-amino-5-((R)-3-(5-((R)-2-(3-aminopropanamido)-2-carboxyethyl)-2,3-dihydroxyphenylthio)-1-(carboxymethylamino)-1-oxopropan-2-ylamino)-5-oxopentanoic acid. (TIF) [file pone.0064475.s003.tif]

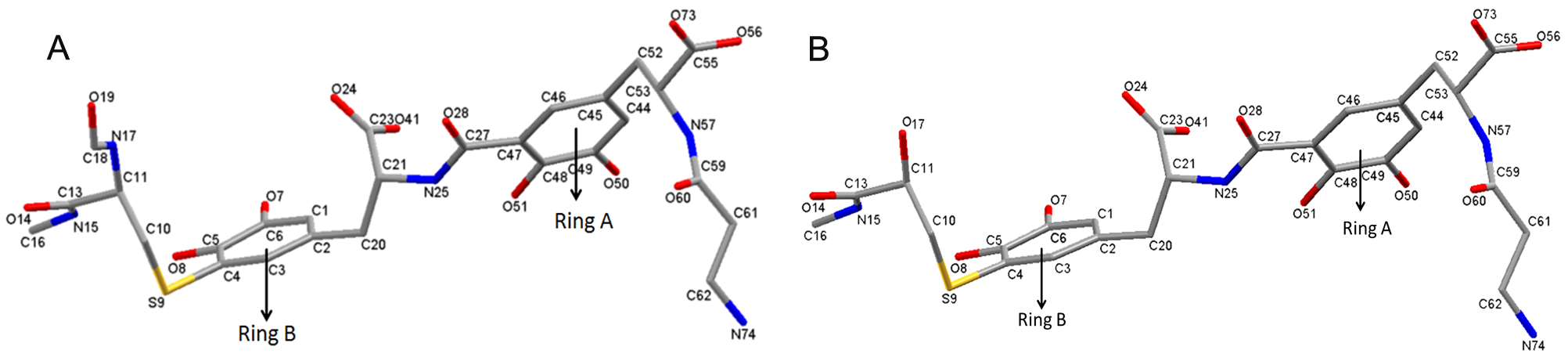

Supplement: Figure S4 — Schematic representations of (A) FSHP_FB and (B) FSHP. (TIF) [file pone.0064475.s004.tif]

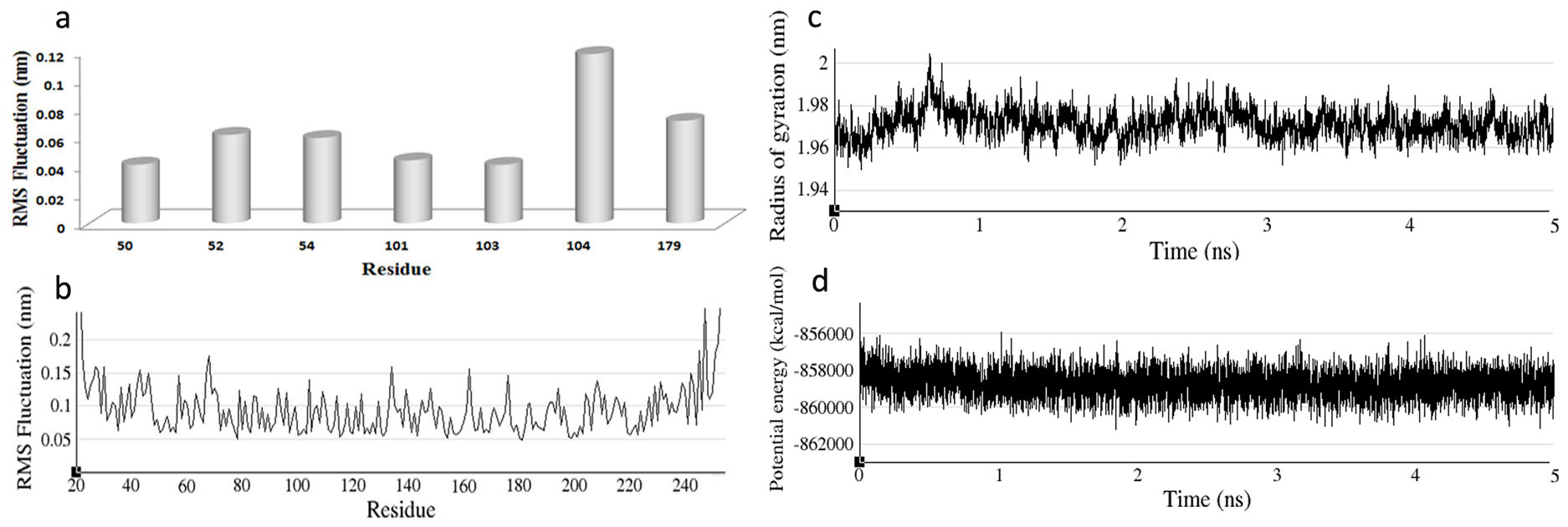

Supplement: Figure S5 — Analysis of MD trajectory. (a) Histogram showing the RMSF values for BSRs. (b) RMSF plot showing residue-wise fluctuations of hFSHR (c) Radius of gyration (d) Potential energy. (TIF) [file pone.0064475.s005.tif]
