# Supplementary material for: In Silico Study on Binding Specificity of Gonadotropins and Their Receptors: Design of a Novel and Selective Peptidomimetic for Human Follicle Stimulating Hormone Receptor
Source: PLoS One. 2013 May 20;8(5):e64475. doi: 10.1371/journal.pone.0064475 (PMC3659097; doi:10.1371/journal.pone.0064475)
Supplement: Table S1 — SAVES results for structural validation of hLHR-LH model. (DOC) [file pone.0064475.s006.doc]

**Table S1.** SAVES results for structural validation of hLHR-LH model

| **Method** | **Result** | **Comment** |
| --- | --- | --- |
| PROCHECK | 74.5% (Core)+23.3%(allowed) | Stereo chemical quality of the protein structure |
| Verify_3D | 82.60% | Compatibility of 3D model with its 1D sequence |
| ERRAT | 69.474 | Overall quality factor of protein structure |
