# Supplementary material for: In Silico Study on Binding Specificity of Gonadotropins and Their Receptors: Design of a Novel and Selective Peptidomimetic for Human Follicle Stimulating Hormone Receptor
Source: PLoS One. 2013 May 20;8(5):e64475. doi: 10.1371/journal.pone.0064475 (PMC3659097; doi:10.1371/journal.pone.0064475)
Supplement: Table S2 — Interactions between MMs02514408 and hFSHR in the docked complexes. (DOC) [file pone.0064475.s007.doc]

**Table S2. Interactions between MMs02514408 and hFSHR in the docked complexes**

| **GOLD docking*** | | | **Glide docking^#^** | | |
| --- | --- | --- | --- | --- | --- |
| **hFSHR^∞^** | **MMs02514408** | **Interaction** | **hFSHR^∞^** | **MMs02514408** | **Interaction** |
| *50E(Oε_2_)* | N61 | Electrostatic | *50E(Oε_2_)* | N66 | Electrostatic |
| *101R(Nη_2_)* | N19 | H bond | *104K(Nζ)* | O8 | H bond |
| *101R(Nη_2_)* | O32 | H bond | *104K(Nζ)* | O21 | H bond |
| *104K(Nζ)* | O8 | H bond | *104K(Nζ)* | O59 | Electrostatic |
| *52R(Nε)* | O27 | Electrostatic | *101R(Nη_1_)* | Phenyl ring | Cation-π |
| *103E(Oε_1_)* | O7 | H bond | *103E(Oε_1_)* | O8 | H-bond |
| 76E(Oε_2_) | N19 | H bond | *103E(Oε_1_)* | N15 | H-bond |
| 78S(Oγ) | O18 | H bond | 76E(Oε_1_) | N66 | Electrostatic |
| Q79(Nε_2_) | O59 | H bond | 76E(Oε_2_) | O7 | H-Bond |
| Q79(Nε_2_) | O18 | H bond | 78S(Oγ) | O8 | H- Bond |
| 128S(Oγ) | O7 | H bond | 78S(Oγ) | O7 | H-Bond |
| D150(Oδ) | N66 | H bond | 129N (Oδ_1_) | N61 | H-Bond |
|  |  |  | 129N (Oδ_1_) | O59 | H-Bond |
|  |  |  | 128S(Oγ) | O18 | H-Bond |

**^*^**GoldScore = 84.185.

^#^XP GlideScore = -6.401 Kcal/mol.

**^∞^**BSRs are italicised.
