# Supplementary material for: In Silico Study on Binding Specificity of Gonadotropins and Their Receptors: Design of a Novel and Selective Peptidomimetic for Human Follicle Stimulating Hormone Receptor
Source: PLoS One. 2013 May 20;8(5):e64475. doi: 10.1371/journal.pone.0064475 (PMC3659097; doi:10.1371/journal.pone.0064475)
Supplement: Table S3 — Interactions between FSHP_FB and FSHR in the docked complexes. (DOC) [file pone.0064475.s008.doc]

**Table S3. Interactions between FSHP_FB and FSHR in the docked complexes**

| **GOLD docking*** | | | **Glide docking^#^** | | |
| --- | --- | --- | --- | --- | --- |
| **hFSHR^∞^** | **FSHP_FB**^ƫ^ | **Interaction** | **hFSHR^∞^** | **FSHP_FB**^ƫ^ | **Interaction** |
| *50E(Oε_2_)* | N74 | Electrostatic | *50E(Oε_2_)* | N74 | Electrostatic |
| *104K(Nζ)* | O28 | H bond | *104K(Nζ)* | O51; O24 | Electrostatic |
| *101R(Nη_2_)* | O24;O41 | Electrostatic | *179K(Nζ)* | O14 | H bond |
| *101R(Nη_2_)* | Phenyl ring-A | Cation-π | *101R(Nη_2_)* | Phenyl ring-A | Cation-π |
| *52R(Nε)* | N74 | Electrostatic | *103E(Oε_1_)* | N25 | H bond |
| 76E(Oε_1_) | N57 | H bond | *103E(Oε_1_)* | O51 | H bond |
| 76E(Oε_2_) | N57 | H bond | 76E(Oε_1_) | N74 | Electrostatic |
| 78S(Oγ) | O28 | H bond | 78S(Oγ) | O50 | H bond |
| 128S(Oγ) | O7 | H bond | 78S(Oγ) | O51 | H bond |
| 152Q(Nε_2_) | O8 | H bond | N129(Oδ_1_) | O7 | H bond |
| D150(Oδ_1_) | N17 | H bond | N129(Oδ_1_) | O8 | H bond |
| D150(Oδ_1_) | N15 | H bond | 152Q(Nε_2_) | S9 | H bond |
|  |  |  | 153D(Oδ_2_) | O8 | H bond |
|  |  |  | 153D(Oδ_2_) | N15 | H bond |

*GoldScore = 78.52.

^#^XP GlideScore = -7.83 Kcal/mol; EModel = -67.44; Interaction energy = 180.86 Kcal/mol. **^∞^**BSRs are italicised.

^ƫ^ Refer to Figure S4A for structural details.
