# Supplementary material for: In Silico Study on Binding Specificity of Gonadotropins and Their Receptors: Design of a Novel and Selective Peptidomimetic for Human Follicle Stimulating Hormone Receptor
Source: PLoS One. 2013 May 20;8(5):e64475. doi: 10.1371/journal.pone.0064475 (PMC3659097; doi:10.1371/journal.pone.0064475)
Supplement: Table S4 — Compounds with their experimental IC50 values and Glide Scores on binding with hFSHR. (DOC) [file pone.0064475.s009.doc]

**Table S4. Compounds with their experimental IC50 values and Glide Scores on binding with hFSHR**

| **S.No.** | **Compound*** | **hFSHR Binding** | | | **Glide Score (Kcal/Mol)** |
| --- | --- | --- | --- | --- | --- |
|  |  | **IC_50_ (μM)*** | | **log IC_50_** |  |
| **Actives** | | | | | |
|  | 1 | | 3.0 ± 0.29 | 0.477 | -3.973 |
|  | 2 | | 1.2 ± 0.06 | 0.079 | -4.909 |
|  | 3 | | 4.0 ± 0.25 | 0.602 | -3.923 |
|  | 8 | | 2.5 ± 0.11 | 0.397 | -5.608 |
|  | 14 | | 2.0 ± 0.21 | 0.301 | -4.320 |
|  | 15 | | 6.2 ± 0.35 | 0.792 | -4.215 |
|  | 17 | | 2.4 ± 0.19 | 0.380 | -4.984 |
|  | 18 | | 2.2 ± 0.09 | 0.342 | -5.288 |
|  | 19 | | 5.9 ± 0.26 | 0.770 | -4.781 |
|  | 20 | | 9.1 ± 0.56 | 0.959 | -4.668 |
|  | 21 | | 6.9 ± 0.26 | 0.838 | -4.431 |
|  | 22 | | 1.3 ± 0.08 | 0.113 | -4.170 |
|  | 25 | | 53% @10μM | 1.000 | -4.010 |
|  | 26 | | 1.1 ± 0.04 | 0.041 | -3.523 |
|  | 28 | | 1.5 ± 0.05 | 0.176 | -4.710 |
|  | 29 | | 3.7 ± 0.71 | 0.568 | -4.062 |
|  | 31 | | 4.1 ± 0.29 | 0.612 | -4.291 |
|  | 32 | | 4.8 ± 0.26 | 0.681 | -4.708 |
|  | 35 | | 2.9 ± 0.09 | 0.462 | -4.232 |
|  | 37 | | 3.5 ± 0.17 | 0.544 | -3.225 |
|  | 38 | | 6.9 ± 0.65 | 0.838 | -4.257 |
|  | 39 | | 2.0 ± 0.29 | 0.301 | -4.250 |
|  | 41 | | 1.6 ± 0.06 | 0.204 | -3.972 |
|  | 42 | | 4.1 ± 0.2 | 0.612 | -3.940 |
|  | 47 | | 3.2 ± 0.2 | 0.505 | -4.193 |
|  | 49 | | 1.9 ± 0.7 | 0.278 | -3.819 |
|  | 50 | | 2.5 ± 0.1 | 0.397 | -4.038 |
| **Inactives** | | | | | |
|  | 4 | | NI @ 30μM (40)^a^ | 1.602 | -2.179 |
|  | 5 | | NI @ 30μM (40)^a^ | 1.602 | -4.499 |
|  | 6 | | NI @ 30μM (40) ^a^ | 1.602 | -3.868 |
|  | 7 | | NI @ 30μM (40)^a^ | 1.602 | -3.199 |
|  | 9 | | 23 ± 0.9 | 1.361 | -3.915 |
|  | 10 | | 20 ± 0.53 | 1.301 | -3.633 |
|  | 11 | | 34 ± 0.9 | 1.531 | -3.164 |
|  | 12 | | 41 ± 1.24 | 1.612 | -3.370 |
|  | 13 | | 16 ± 0.74 | 1.204 | -4.895 |
|  | 16 | | 48% @ 100μM (110)^a^ | 2.041 | -3.586 |
|  | 23 | | NI @ 10μM (20)^a^ | 1.301 | -4.236 |
|  | 24 | | 38% @10μM (19.6) ^a^ | 1.292 | -3.089 |
|  | 27 | | NI @30μM (40)^a^ | 1.602 | -3.366 |
|  | 30 | | 28% @10μM (19.7)^a^ | 1.294 | -4.805 |
|  | 33 | | 30% @ 10μM (19.7)^a^ | 1.294 | -4.202 |
|  | 34 | | 42% @ 10μM (19.5)^a^ | 1.290 | -3.709 |
|  | 36 | | 26% @ 10μM (19.7)^a^ | 1.294 | -3.397 |
|  | 40 | | 68 ± 11.2 | 1.832 | -2.835 |
|  | 43 | | 38% @ 10μM (19.6)^a^ | 1.292 | -3.579 |
|  | 44 | | 17% @ 10μM (19.8)^a^ | 1.296 | -2.168 |
|  | 45 | | 15% @ 10μM (19.8)^a^ | 1.296 | -3.077 |
|  | 46 | | 38% @ 10μM (19.6)^a^ | 1.292 | -4.455 |
|  | 48 | | NI @ 30μM (40)^a^ | 1.602 | -4.074 |

* The compounds and their experimentally determined IC50 values are taken from the report of Wrobel et al. (2001). NI represents no inhibition. ^a^In these cases, the exact IC50 values were not available. To obtain a quantitative measure, a penalty of 10 (chosen arbitrarily) was added to the tested concentration and the percent inhibition was subtracted from this value.
